# Developmental origin underlies evolutionary rate variation across the placental skull

**DOI:** 10.1098/rstb.2022.0083

**Published:** 2023-07-03

**Authors:** Anjali Goswami, Eve Noirault, Ellen J. Coombs, Julien Clavel, Anne-Claire Fabre, Thomas J. D. Halliday, Morgan Churchill, Abigail Curtis, Akinobu Watanabe, Nancy B. Simmons, Brian L. Beatty, Jonathan H. Geisler, David L. Fox, Ryan N. Felice

**Affiliations:** ^1^ Department of Life Sciences, Natural History Museum, London SW7 5BD, UK; ^2^ Department of Genetics, Evolution, and Environment, University College London, London WC1E 6BT, UK; ^3^ Centre for Integrative Anatomy, Department of Cell and Developmental Biology, University College London, London WC1E 6BT, UK; ^4^ Department of Vertebrate Zoology, National Museum of Natural History, Smithsonian Institution, Washington, DC 20013, USA; ^5^ Department of Paleobiology, National Museum of Natural History, Smithsonian Institution, Washington, DC 20013, USA; ^6^ Université Lyon, Université Claude Bernard Lyon 1, CNRS, ENTPE, UMR 5023 LEHNA, 69622 Villeurbanne, France; ^7^ Naturhistorisches Museum Bern, 3005 Bern, Switzerland; ^8^ Institute of Ecology and Evolution, University of Bern, 3012 Bern, Switzerland; ^9^ School of Geography, Earth and Environmental Sciences, University of Birmingham, Birmingham B15 2TT, UK; ^10^ Department of Biology, University of Wisconsin Oshkosh, Oshkosh, WI 54901, USA; ^11^ Department of Biology, University of Washington, Seattle, WA 98195, USA; ^12^ Department of Anatomy, College of Osteopathic Medicine, New York Institute of Technology, Old Westbury, NY 11568, USA; ^13^ Division of Paleontology, American Museum of Natural History, New York, NY 10024, USA; ^14^ Department of Mammalogy, Division of Vertebrate Zoology, American Museum of Natural History, New York, NY 10024, USA; ^15^ Department of Earth and Environmental Sciences, University of Minnesota, Minneapolis, MN 55455, USA

**Keywords:** skull evolution, morphometrics, cranial neural crest, development, ecology

## Abstract

The placental skull has evolved into myriad forms, from longirostrine whales to globular primates, and with a diverse array of appendages from antlers to tusks. This disparity has recently been studied from the perspective of the whole skull, but the skull is composed of numerous elements that have distinct developmental origins and varied functions. Here, we assess the evolution of the skull's major skeletal elements, decomposed into 17 individual regions. Using a high-dimensional morphometric approach for a dataset of 322 living and extinct eutherians (placental mammals and their stem relatives), we quantify patterns of variation and estimate phylogenetic, allometric and ecological signal across the skull. We further compare rates of evolution across ecological categories and ordinal-level clades and reconstruct rates of evolution along lineages and through time to assess whether developmental origin or function discriminate the evolutionary trajectories of individual cranial elements. Our results demonstrate distinct macroevolutionary patterns across cranial elements that reflect the ecological adaptations of major clades. Elements derived from neural crest show the fastest rates of evolution, but ecological signal is equally pronounced in bones derived from neural crest and paraxial mesoderm, suggesting that developmental origin may influence evolutionary tempo, but not capacity for specialisation.

This article is part of the theme issue ‘The mammalian skull: development, structure and function’.

## 1. Introduction

The diversification of mammals following the end-Cretaceous mass extinction represents one of the best examples of adaptive radiation captured by the fossil record [1,2]. Within a few million years of the mass extinction, mammals had evolved larger body sizes than observed through the whole of the Mesozoic and diversified into numerous specialist ecological niches, including the first large-bodied mammalian herbivores [3–7]. By the early Eocene, mammals had even taken to the seas and skies [8–10]. This diversification and specialization involved extreme modification of the skeleton, including the cranium [2,11,12]. Yet, the skull is not composed of a single element, but is rather a composite structure formed from numerous elements with different developmental origins and with multiple functions [13–18]. Previous studies in diverse vertebrate clades have explored how different cranial regions can display divergent macroevolutionary dynamics and varying associations with ecological, developmental and life-history factors, from diet and locomotion to reproductive strategy [14,19–24]. The effect of these diverse influences on cranial shape may be reflected in patterns of cranial modularity, wherein the skull can be partitioned into semi-autonomous subunits that are tightly integrated internally but have weaker covariation with other regions [25–32]. Numerous studies have quantified modularity and integration in the vertebrate cranium, assessing patterns, causes and consequences of its modular organization [25,28,33–42]. Differences in methodologies across studies complicate agreement on a single pattern of cranial modularity for any dataset. Ultimately, it is likely that there exists a hierarchical organization of the cranium where a broadly facial–neurocranial division can be decomposed into smaller modules, e.g. rostrum, orbit, vault, base, etc. themselves composed of multiple individual elements or structures [25,31,43,44]. While cranial modules are often described in terms of function, developmental patterning has long been considered a primary cause of cranial integration and modularity [27,28,45,46], providing a direct link between associations among phenotypic traits and their developmental origins.

**Figure 1.**
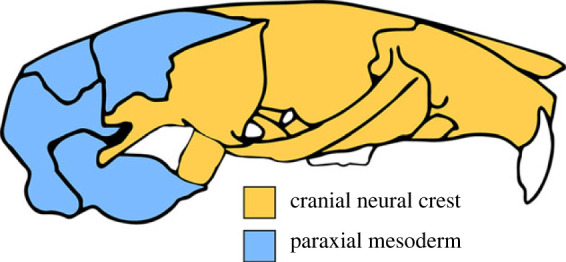
Developmental origins of cranial elements in the placental skull. Modified from Piekarski *et al.* [48].

Two distinct cranial mesenchymal stem cell populations give rise to the bones of the skull: the cranial neural crest (CNC) and the paraxial mesoderm (PM) [47,48]. The cranial neural crest is derived from the embryonic ectoderm and forms the anterior skull, while the paraxial mesoderm generally forms the posterior bones of the skull and is derived from the embryonic mesoderm [15,16,18,47–49]. The boundary between CNC- and PM-derived tissues has been subject to extensive debate, but in mammals roughly corresponds to the frontal–parietal suture on the dorsal skull and the basisphenoid–presphenoid suture on the ventral skull [18,47,49,50]. The structures of the face, middle ear, mandible, zygomatic arch and palate, including the pterygoids, are formed from CNC cells, while the posterior vault and occiput are PM-derived (figure 1). Elements in the boundary region, such as the bones of the sphenoid complex, may have contributions from both stem cell populations [49].

It has been hypothesized that elements formed from CNC have a greater capacity for variation, reflecting the multipotency of this cell population [51–55]. In particular, the neural crest has been implicated in the evolution of morphological novelties, such as the vertebrate head and jaw [51,56]. The impact of neural cell multipotency has been hypothesized to extend beyond novel or multiple cell types and structures to providing increased variation of traits, including those under both natural and sexual selection [57]. Analysis of domesticated animals has also identified higher variation in neural crest-derived regions of the skull [58,59], and the repeated evolution of specific phenotypes in domesticated animals has also been traced to neural crest cell behaviour [60]. Combined, these studies suggest that neural crest-derived regions may have greater evolutionary capacity than those derived from paraxial mesoderm, though this hypothesis is largely untested outside of domesticated systems.

Recent macroevolutionary studies of amphibians, reptiles and birds have demonstrated that the fastest-evolving cranial regions are those derived from CNC [14,19–21], and that these fast-evolving regions are generally located in either the suspensorium, including the varied elements associated with the jaw articulation or the anterior face, but rarely both. Amphibians and squamates display variation concentrated in the suspensorium [19–21], while birds show the highest variation in the rostrum [14]. Birds overall showed the fastest evolution in regions that incorporate cranial neural crest, in particular its anterior mandibular stream [14]. Other archosaurs (crocodiles and non-avian dinosaurs) show a more diffuse pattern spanning both the anterior face and suspensorium (which is derived from the posterior mandibular stream of the CNC), as well as the posterior vault in dinosaurs, a region that is heavily ornamented in some clades [61,62]. Some studies of mammals and birds have suggested that CNC-derived tissues have exceptional capacity for generating variation [60,63–65], but there is also evidence that cranial regions that include both CNC- and PM-derived tissues, such as the vault, show lower integration and higher disparity [66]. While these effects may be expected to impact the pace of evolution and amount of disparity equally, rates of evolution and disparity do not necessarily correspond, and areas with low disparity may yet display fast rates of evolution, and vice versa [67,68].

In addition to developmental complexity and its potential consequences, the skull performs numerous unrelated functions, from prey capture and food processing, to housing the brain and sensory structures, to bearing ornaments and appendages for combat and defence, to facilitating locomotion, such as in burrowing by head lift digging or tooth digging [25,69–80]. Thus, different cranial regions may be expected to display different evolutionary patterns, depending on the ecology and behaviours of individual clades or lineages. For example, lizards show a strong association between diet and the shape of the rostrum, while in snakes, a stronger association with diet is observed in the shape of the bones of the suspensorium, responsible for controlling gape [19].

Distinguishing the impacts of developmental patterning and of function on the evolutionary dynamics of any cranial region or individual element is complicated by overlapping hypotheses for their respective influences [25,46], e.g. the elements of the rostrum are also those formed exclusively from CNC cells, while those of the cranial base are exclusively formed from PM. Further complicating discrimination of these effects are functional regions where both cell populations contribute to the formation of elements, such as the vault and orbit. As these regions are also intermediate between the anterior and posterior regions of the skull, they may also be more likely to experience conflicting or competing functional pressures. Previous studies of the evolutionary dynamics of cranial modules have suggested that these developmentally (and likely functionally) complex regions may show lower integration and higher variation in birds and mammals [14,25,66,67], although this is not supported in other vertebrate clades [19,22,81].

Here, we use a large three-dimensional dataset of 322 living and extinct eutherian mammals to quantify and compare macroevolutionary dynamics across the elements forming the cranium. Our sample ranges from the smallest living mammal, the bumblebee bat (*Craseonycteris thonglongyai*), to the largest, the blue whale (*Balaenoptera musculus*). Using a high-dimensional geometric morphometric approach to discriminate 17 cranial elements (representing 12 individual cranial bones, as some are subdivided into different aspects or structures), we quantify variation, phylogenetic, allometric and ecological signals across the skull and reconstruct the tempo of evolution and disparity for each region through time. Although these elements certainly form higher-level modules [25,34], we treat them here independently to maximize the ability to link observed patterns to developmental origin and function. With this high-resolution dataset that is more capable of discriminating patterns for each cranial element compared to previous approaches (e.g. landmarks only or lengths), we assess whether elements of the mammalian cranium that share a common developmental origin or function also share a common pattern of evolution. In particular, we assess the hypothesis that elements formed from CNC cells show greater variation, faster rates of evolution, and stronger ecological signal, relative to those derived from the PM.

## 2. Methods

### (a) Material and methods

#### (i) Specimens and morphometric data

**Figure 2.**
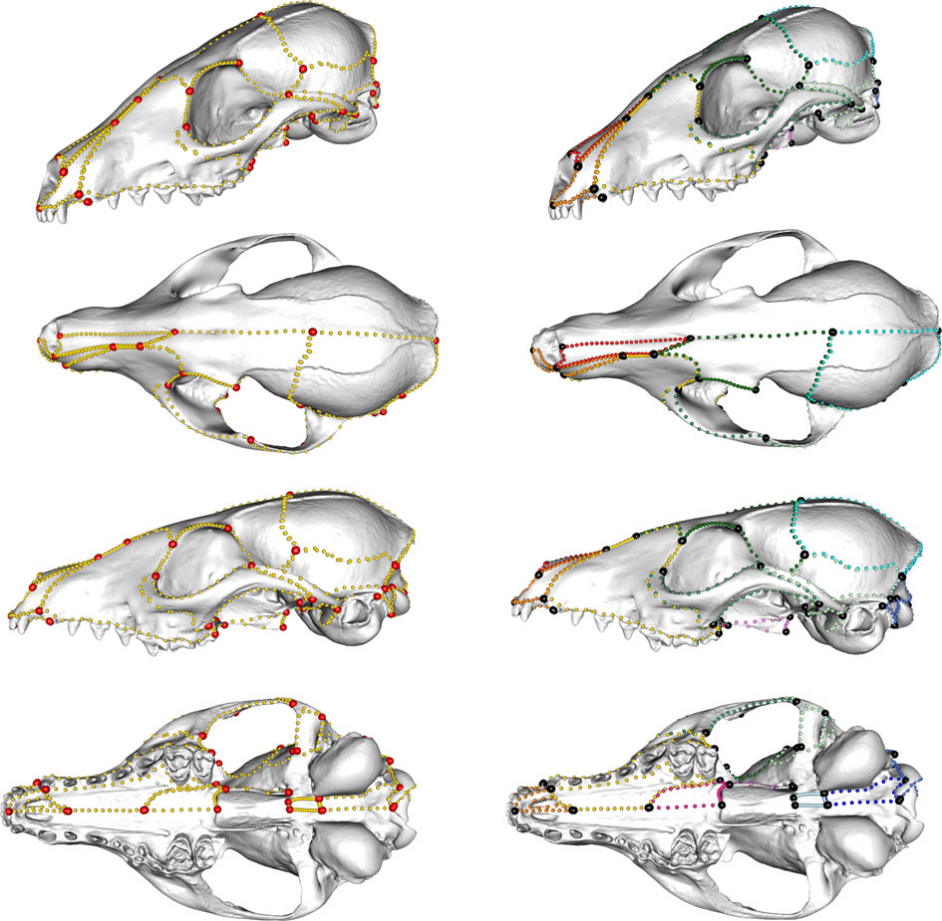
Morphometric data shown on *Vulpes pallida* in oblique, dorsal, lateral and ventral views, from top to bottom. Left: Cranial landmarks (red) and semi-landmarks (gold). Right: Data coloured by cranial elements: nasal (red), premaxilla (orange), maxilla (yellow), jugal (green), frontal (dark green), squamosal (light green), pterygoid (light purple), palatine (pink), parietal (sky blue), occipital (dark blue) and basisphenoid (light blue)*.* Note that the premaxilla, maxilla, squamosal and occipital are further separated into smaller regions in analyses. Regions and morphometric data are detailed further in electronic supplementary material, table S2.

Our dataset samples 322 crown and stem placental mammals, comprising 207 extant and 115 extinct species (electronic supplementary material, table S1), from a recently published analysis [2]. Each species is represented by a single adult specimen. Sixty-six three-dimensional landmarks and 69 semi-landmark curves were collected for the left side of the skull using Stratovan Checkpoint (Stratovan, Davis, CA, USA) (figure 2; electronic supplementary material, table S2). A total of 754 three-dimensional landmarks and semi-landmarks were then imported into R [82] for analysis and curves were resampled to a common number of semi-landmarks using the ‘SURGE’ package [83]. Specimens were selected for completeness, but some structures were missing or incomplete from preservation (as opposed to biologically absent). As information on sex is not known with certainty for most fossil specimens and is lacking for many extant specimens, variation associated with sex could not be accounted for in this study. Out of 322 specimens, 102 were missing pterygoids, 49 were missing jugals (of which eight were biologically absent and thus not reconstructed), 33 were missing basisphenoids (largely because these were inaccessible in surface scans due to overlapping palatines) and 22 were missing the zygomatic process of the squamosal. Missing structures were estimated using *fixLMtps*, which uses weighted averages from the three most morphologically similar and complete configurations to estimate missing landmarks, in the R package ‘Morpho’ v.2.9 [84], after which they were slid to minimize bending energy. Landmarks and semi-landmarks for biologically absent elements were moved to a single landmark position, as described in Bardua *et al.* [22,85]. Morphometric data were mirrored across the midline plane to create bilaterally symmetrical landmark configurations, and then registered with Generalised Procrustes Analysis in the R package ‘geomorph’ v.4.04 [86]. Mirrored (right-side) data were then removed to reduce dimensionality. Finally, the full morphometric dataset was separated into the following 17 cranial regions for analysis: dorsal premaxilla, ventral premaxilla, nasal, dorsal maxilla, ventral maxilla, palatine, pterygoid, jugal, frontal, parietal, zygomatic region of the squamosal, glenoid fossa of the squamosal (jaw articulation), vault region of the squamosal, supraoccipital, occipital condyles, basioccipital and basisphenoid. These regions were grouped by stem cell origin (cranial neural crest or paraxial mesoderm, as indicated in table 1), in further analyses, as detailed below.

**Table 1.** Analysis of phylogenetic (*K*_mult_) and allometric signal (*Z*-score), disparity, and evolutionary rate in 17 cranial regions, quantified across 322 living and extinct placental mammals. Asterisk indicates significance at *p* < 0.01 level.

	phylogeny	allometry	disparity	rate (×10^−7^)
**neural crest-derived elements**				
nasal	0.820*	4.41*	0.0055	5.810
premaxilla (d)	0.704*	4.77*	0.0045	6.285
premaxilla (v)	0.693*	3.77*	0.0029	8.588
maxilla (d)	0.554*	4.71*	0.0048	5.416
maxilla (v)	0.565*	4.04*	0.0027	4.905
palatine	0.406*	2.61*	0.0011	3.883
jugal	0.380*	4.10*	0.0025	7.021
frontal	0.473*	5.24*	0.0047	6.362
squamosal (v)	0.523*	5.21*	0.0015	4.202
squamosal (z)	0.475*	1.99	0.0012	5.624
glenoid fossa	0.521*	2.86*	0.0007	4.219
pterygoid	0.359*	2.40	0.0004	3.877
**paraxial mesoderm-derived elements**				
parietal	0.493*	5.99*	0.0034	5.499
supraoccipital	0.499*	5.76*	0.0027	4.005
occipital condyle	0.545*	4.39*	0.0008	3.812
basioccipital	0.433*	3.78*	0.0006	2.269
basisphenoid	0.328*	2.72*	0.0006	2.549

#### (ii) Phylogenetic and ecological data

Data on diet and locomotion were collected from the published literature using palaeoecological reconstructions for fossil taxa as detailed in Goswami *et al.* [2] and electronic supplementary material, table S1. In the absence of a well-resolved phylogenetic hypothesis that samples all living and extinct taxa in our dataset, we constructed composite trees from molecular and morphological analyses by grafting fossil taxa onto a recent species-level molecular analysis of placental mammal relationships [87], as described in Goswami *et al.* [2]. Specifically, we binned the posterior distribution of dated trees from that study by estimated origin of placental mammals and randomly selected one tree from the bin ranging from 80–85 Ma, consistent with the most recent analyses [88]. The selected tree (placental divergence estimate = 80.3 Ma) was then used as the base tree for addition of our sampled fossil taxa based on recent morphological analyses, as detailed in Goswami *et al.* [2]. Most of the fossil taxa in our sample are reasonably well resolved in terms of phylogenetic affiliations, with remaining uncertainty largely involving within-group relationships that should have little impact on model estimations at our level of sampling. We initially generated three alternative composite topologies that capture the major points of uncertainty in the relationships of early placentals, such as placement of cimolestids and ‘amblyopods’ [89]. However, our recent whole-skull analyses [2], conducted across a sample of 1800 trees that vary in topology and divergence estimates, demonstrated that this uncertainty in topology had little impact on results, and thus we focus on a single phylogenetic framework here to allow for meaningful comparison across cranial regions. Nonetheless, it is important to remember that uncertainty in the phylogenetic positions and divergence estimates can impact results and that this issue will only be resolved through continuing dedicated systematic analysis that includes broad sampling of the excellent Cenozoic mammal fossil record.

### (b) Macroevolutionary analyses

To examine the overall pattern of variation in individual cranial regions across placentals, we conducted principal components analyses using Procrustes-aligned three-dimensional data. We further conducted phylogenetic principal components analysis for use in the branch-specific rate analyses described below. We quantified phylogenetic signal using *K*_mult_, disparity as Procrustes variance, and evolutionary allometry using Procrustes ANOVA of shape (Procrustes coordinates) against log(centroid size), incorporating phylogeny under a Brownian motion model of evoution, as implemented in the R package ‘geomorph’ using *procD.pgls*. We also calculated evolutionary rates for each cranial region under a Brownian motion model using the *compare.multi.evol.rates* function in ‘geomorph’ (with 999 iterations for significance testing), in order to facilitate direct comparison of rates across regions [90,91]. We then used the estimates of disparity and evolutionary rate to assess whether elements of cranial neural crest or paraxial mesoderm origin differ significantly in evolutionary capacity, using non-parametric analyses of variance.

We further assessed the association of life-history and ecological traits with shape variation and evolutionary rate for each cranial region with multivariate phylogenetic regressions. We conducted type II phylogenetic MANOVAs with Pagel's lambda by penalised likelihood on the Procrustes coordinates with log centroid size, locomotion, and diet as predictors, using the functions ‘mvgls’ and ‘manova.gls’ as implemented in the R package ‘mvMORPH’ v.1.16 [92]. Pillai's statistic and 1000 permutations were used to assess significance. Note that Pagel's lambda corresponds to fitting a phylogenetic mixed model which can provide increased flexibility in estimating the error structure and allows for departures from a Brownian motion model. We then estimated ancestral states for locomotion and diet using stochastic character mapping with an ‘All Rates Different’ (ARD) model, which allows different transition rates between character states, in the ‘phytools’ v.0.7–70 package [93]. We then used a state-specific Brownian motion (BM) model in ‘mvMORPH’ to estimate rates of evolution for each locomotory and dietary state for each cranial region. Model fitting jointly estimated measurement error and intraspecific variation, which is again flexible to departures from Brownian motion.

For each cranial region, we further assessed 10 alternative evolutionary models (variable- and single-rate models for Brownian motion, single-optimum Ornstein-Uhlenbeck, and variable- and single-rate BM models with lambda, kappa or delta tree transformations). A lambda tree transformation captures fit to phylogenetic structure, a kappa tree transformation reflects punctuational processes, and a delta transformation equates to an early burst model. Each analysis used phylogenetic PC scores representing 95% of the total variation in the dataset (ranging from three PCs in the ventral premaxilla to 14 for the dorsal maxilla) and a reversible-jump Markov Chain Monte Carlo (MCMC) algorithm implemented in BayesTraits v.3 [94]. Models ran for 500 000 000 iterations, and convergence of the chains was assessed using Gelman and Rubin's convergence diagnostic implemented in the R package ‘coda’ v.0.19-3 [95]. Bayes Factor (BF) was used to compare models and identify the best supported model. For the best supported model, we plotted rates of evolution on branches to visualize rate variation across the phylogeny. We then binned rates between successive nodes in the tree by averaging across coexisting lineages in 1 Myr time bins and plotted their pattern through time, grouped by stem cell origin. We further extracted rates for the terminal branches for each cranial region and plotted them by clade to assess differences in mean rate across clades.

## 3. Results

### (a) Cranial variation

**Figure 3.**
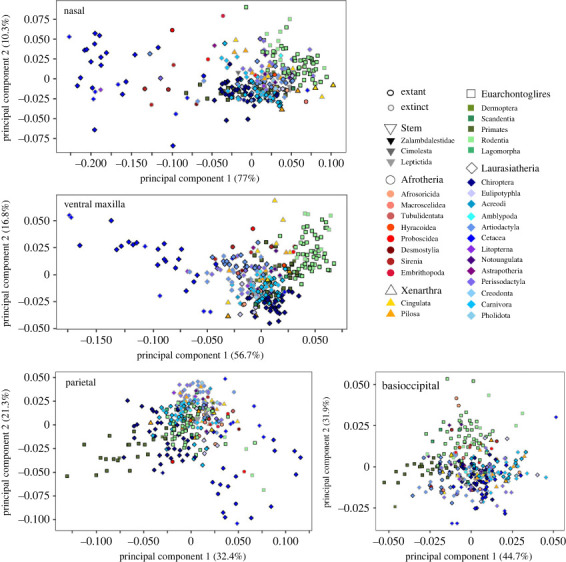
Principal component analyses of four representative cranial regions: nasal, ventral maxilla, parietal and basioccipital. PCs 1–4 for all regions provided in electronic supplementary material, figure S1, with interactive PCAs available at https://github.com/anjgoswami/Goswami_et_al_Placental_evolution_2022.

The distribution of species along the primary principal component axes varies widely across different cranial elements (figure 3; electronic supplementary material, figure S1), demonstrating that macroevolutionary patterns are not uniform across the placental cranium. Some elements show similarity in patterns; for example, there was extensive overlap across all placental mammal orders in the shape of the elements forming the zygomatic arch, as well as the palatine and basisphenoid. Whales were highly differentiated from other placentals in the nasals, premaxilla, maxilla and, to a lesser extent, in the frontal, parietal, occipital condyles, supraoccipital and pterygoids. Many afrotherian clades were clearly differentiated from other placentals in the shape of the nasals, premaxilla and maxilla. Rodents were differentiated from other placentals in the shape of the maxilla, vault portion of squamosal, basioccipital and glenoid fossa. Primates stood out from other placentals in the shape of the frontal, parietal and, to a lesser extent, basioccipital. Bats also are differentiated from other placentals in the shape of their ventral maxilla and premaxilla, palatine, pterygoid, parietal and, to a lesser extent, nasal and supraoccipital. Bats are also well differentiated from other placentals on PC3 in the premaxilla, jugal, occipital condyle and, to a lesser extent, the regions of the squamosal (electronic supplementary material, figure S1).

Disparity (table 1) is highest in the nasals and lowest in the pterygoids. However, it is important to note that the pterygoids were missing and thus estimated in approximately one-third of specimens (described in Methods), which may underestimate their true disparity. However, additional analyses removing specimens with missing pteryoids (102 specimens), as well as another analysis removing specimens with any missing elements at all (138 specimens), demonstrates that this has no impact on results (electronic supplementary material, table S3). While the majority of high disparity elements are formed from CNC cells (nasal, maxilla, frontal, premaxilla; table 1), there is not a significant difference in disparity between elements or structures formed from CNC and those formed from PM (Mann-Whitney *U* Test, *p* = 0.246), even excluding the pterygoid (*p* = 0.126).

### (b) Correlates of cranial variation

Phylogenetic signal is statistically significant for all cranial elements, but ranges broadly in its magnitude (table 1). Consistent with the distribution of clades in the regional morphospaces (figure 3; electronic supplementary material, figure S1), the strongest phylogenetic signals were observed in the anterior face, particularly the nasals (*K*_mult_ = 0.82), premaxilla (*K*_mult_ = 0.70 and 0.69 for dorsal and ventral aspects, respectively) and maxilla (*K*_mult_ = 0.554 and 0.565, for dorsal and ventral aspects, respectively), with the lowest values observed in the basisphenoid (*K*_mult_ = 0.33), pterygoid (*K*_mult_ = 0.36), jugal (*K*_mult_ = 0.380) and palatine (*K*_mult_ = 0.46) (table 1).

Phylogenetic regressions against size, diet and locomotion showed immense variation across cranial elements (electronic supplementary material, table S4). Size was a significant factor associated with shape in all regions, with the strongest effect sizes for centroid size observed in the vault and occipital, specifically in the parietal, supraoccipital and frontal. Diet was significantly associated with shape variation (at *p* < 0.05 significance level) for the basioccipital, glenoid fossa, dorsal and ventral maxilla, occipital condyles, palatine, parietal, pterygoid, zygomatic process of the squamosal and supraoccipital (electronic supplementary material, table S4). Locomotion was significantly associated with element shape for the glenoid fossa, dorsal maxilla, occipital condyles, dorsal and ventral premaxilla, vault and zygomatic processes of the squamosal and supraoccipital. The only significant interactions between factors were for size and diet in the basioccipital, ventral maxilla, occipital condyles, palatine, pterygoid and supraoccipital. Size and locomotion interact significantly in the shape of the glenoid fossa, occipital condyles and supraoccipital.

Diet had the strongest effect on the shape of the basioccipital and palatine, followed by size (electronic supplementary material, table S4). Size had the greatest effect across all other cranial modules, although the effect of diet was near equal in the pterygoid.

### (c) Rates of cranial evolution

Rates of evolution as calculated under the assumption of a Brownian motion model vary widely across cranial regions, with the highest rate in the ventral premaxilla and the lowest in the basioccipital (table 1; *p*-values of pairwise comparisons in electronic supplementary material, table S5). Rates of evolution are also significantly higher in elements derived from CNC than in those originating from PM (Mann-Whitney *U* test, *p* = 0.0232).

**Figure 4.**
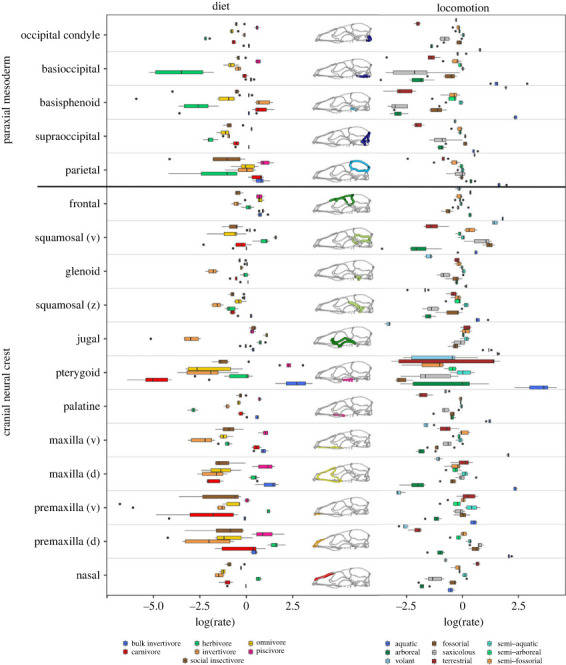
Ecological influences on evolutionary tempo. Rates of evolution in each cranial region, separated by ecological category for diet and locomotion.

Ecological categories that are dominated by whales (e.g. aquatic locomotion, bulk invertivory or piscivory) show high rates in most modules (figure 4). For locomotion, the highest rates are for the aquatic category for all modules except for the basisphenoid and vault region of the squamosal, whereas for diet, bulk invertivores and piscivores show the highest rates in the maxilla, palatine, pterygoid and parietal (figure 4). Piscivores (which include a broader phylogenetic range than does the bulk invertivores group) also show among the highest rates in the occipital condyles and dorsal premaxilla. However, herbivores overall show among the highest rates across the most modules, including the nasal, premaxilla, jugal, glenoid fossa, vault region of the squamosal and basisphenoid. Carnivores show high rates for the maxilla, frontal, parietal and basicranial region, while insectivores show high rates for the parietal and basicranial region, as well as vault region of the squamosal. Finally, omnivores show high rates in the jugal and frontal.

Across locomotory categories other than aquatic, we observed that arboreal taxa show the highest rates for the jugal, while fossorial taxa show high rates in the occipital region and posterior vault (parietal and squamosal; figure 4). Semi-aquatic taxa show high rates in the nasal and premaxilla, while semi-fossorial taxa show high rates in the jugal, premaxilla and zygomatic component of the squamosal. Volant taxa show high rates in the posterior vault and basisphenoid. Terrestrial taxa show high rates only in the premaxilla.

**Figure 5.**
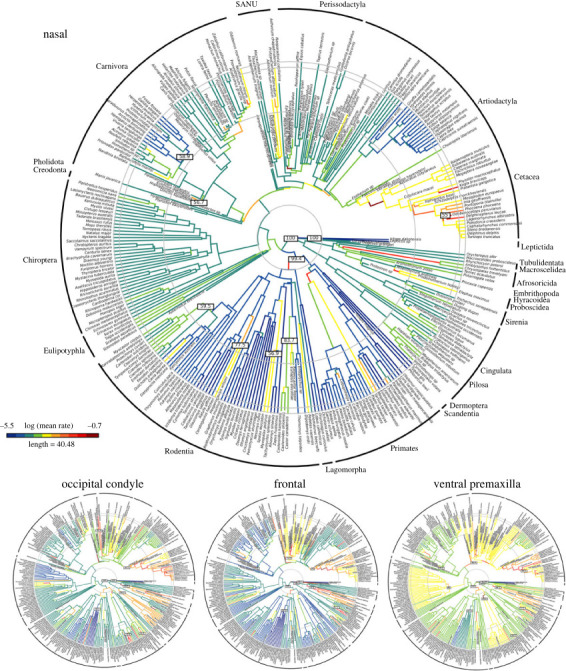
Evolutionary rates per branch for four representative cranial regions. All extracted from analysis with BayesTraits and displaying a variable rates Brownian motion model with a lambda tree transformation. Full results in electronic supplementary material, figure S2.

Branch-specific modelling of evolutionary rates finds that a variable rates Brownian motion model with a lambda tree transformation is best supported for each cranial module with BF > 10 (electronic supplementary material, figure S2), with the exception of a kappa tree transformation being better supported than lambda (still with a variable rates BM model) for the dorsal premaxilla (BF = 3.3). The distribution of evolutionary rates across the tree in each cranial region highlights numerous clade-specific patterns (figure 5; electronic supplementary material, figure S3). Within the facial region, the nasals show the highest rates of change, unsurprisingly, at the base of Neoceti, as well as within odontocete lineages with extensive asymmetry related to echolocation, such as *Kogia* (figure 5). High rates of nasal evolution are also observed at the base of several clades, including Proboscidea, Haplorhini, Rodentia, Odobenidae and Brontotheriidae. Relatively few clades show high rates of evolution in the dorsal premaxilla, but these include the horned rodents, brontotheres and walruses. High rates in the dorsal maxilla are concentrated in cetaceans, as well as in horned rodents and sabre-toothed cats and at the base of lagomorphs, ungulates and perissodactyls (electronic supplementary material, figure S3).

In the zygomatic region, fast rates of evolution for the jugal are also concentrated in cetaceans, but higher rates are observed at the base of rodents and Felidae, and across Afrotheria, pinnipeds, the unusual and extinct South American Native Ungulates (SANUs) [96,97], and some branches of Xenarthra (electronic supplementary material, figure S3). The zygomatic process of the squamosal similarly shows high rates of evolution within cetaceans and proboscideans, and in early branches of Euungulata, Glires, anthropoid primates, brontotheres, nimravid carnivorans, horned rodents, mole-rats, beavers, glyptodonts and golden moles. The glenoid fossa of the squamosal shows the highest rates of evolution in cetaceans, Paenungulata, some extinct sloths, and at the base of artiodactyls, yangochiropteran bats, Glires, anthropoid primates and some rhinolophoid bats.

In the palatal region, the ventral premaxilla shows sustained higher rates in several groups, including Chiroptera, Perissodactyla, SANUs, some artiodactyls, Cetacea, Paenungulata, haplorrhine primates and glyptodonts, as well as at the base of Euarchontoglires (figure 5). The ventral maxilla shows fewer branches with fast rates of evolution limited to specific lineages within cetaceans and the basal branches of anthropoid primates, glyptodonts, anteaters, yangochiropteran bats, sabre-toothed cats and brontotheres (electronic supplementary material, figure S3). The palatine shows higher rates of evolution across Cetacea and Paenungulata, as well as in glyptodonts, walruses and brontotheres, at the base of anthropoids, yangochiropteran bats, Glires, rodents and Euungulata. Unsurprisingly, the unusual pterygoids of cetaceans are reflected in high rates of evolution for that region throughout Cetacea, as well as in pinnipeds, SANUs, perissodactyls, golden moles, proboscideans, mole-rats, soricids, ctenomyid and octodontid rodents, and at the base of Yangochiroptera and Euungulata (electronic supplementary material, figure S3).

Moving to the vault, there are high rates of evolution in the frontal across Cetacea, Perissodactyla, SANUs, Paenungulata, and at the base of rodents (figure 5). There are surprisingly few high rates of frontal evolution observed in artiodactyls, possibly reflecting the lack of landmarks or curves capturing cranial ornaments in this analysis. The parietal showed few fast rates of evolution, which are distributed along individual branches rather than showing sustained high rates across clades (electronic supplementary material, figure S3). These branches include the base of Chiroptera, a few branches at the base of Cetacea and within odontocetes, some within Eulipotyphla and Afrosoricida, and a few branches at the base of rodent clades, including the horned rodents and mole-rats. The vault contribution of the squamosal shows high rates of evolution in cetaceans, suids, xenarthrans, pinnipeds, SANUs, perissodactyls and strepsirrhine primates, at the base of Paenungulata, Glires, rodents, beavers, vampire bats, creodonts and nimravids. The supraoccipital bridges the vault and occipital regions, and high rates in this region are concentrated in Cetacea and Paenungulata, with higher rates also observed in early diverging eutherians (e.g. leptictids; electronic supplementary material, figure S3).

Finally, in the occipital region, the occipital condyles show fast rates of evolution in the cetaceans, paenungulates, pilosan xenarthrans, hominids, vampire bats, nimravids, early diverging pinnipeds, notoungulates and rhinoceroses (figure 5). The basioccipital shows high rates of evolution across placentals, particularly in cetaceans, afrotherians, chiropterans, SANUs, mole-rats and beavers. The basisphenoid, in contrast, shows low to moderate rates of evolution across the tree, with concentrations of higher rates in some cetaceans, afrotherians, glyptodonts and SANUs (electronic supplementary material, figure S3).

Extracting rates for each placental order to compare clades more directly shows clearly that Cetacea dominates the highest rates of evolution in all cranial regions (electronic supplementary material, figure S4). Beyond cetaceans, Afrotheria (particularly paenungulates) and SANUs also show high rates of evolution in most cranial regions. Chiroptera, particularly Yangochiroptera, show fast evolution in specific regions, such as the palate, known to be highly variable in bats [98–100], as well as the parietal and basioccipital (electronic supplementary material, figure S4). The vault in general, and especially the frontal, shows fast evolution across Perissodactyla, reflecting the extreme ornamentation in some clades, such as brontotheres, as well as in the SANU clade Litopterna. Litopterns, similar to whales, have a posteriorly shifted nares and deeply grooved frontal bone (electronic supplementary material, figure S1). The vault portion of the squamosal shows fast rates of evolution across a broad range of placentals, reflecting its highly varied contribution to this structure, which ranges from barely invading the vault region in some bats to forming the majority of the lateral vault in rodents. Finally, unusual extinct taxa, such as sabre-toothed cats, nimravids, SANUs, brontotheres and glyptodonts, frequently display fast rates of evolution in specific regions that exemplify their key characteristics, such as cranial ornaments or elongate teeth, but notable changes in posture are also reflected in rates of regional evolution, such as in the cranial base of hominids (electronic supplementary material, figures S3 and S4).

**Figure 6.**
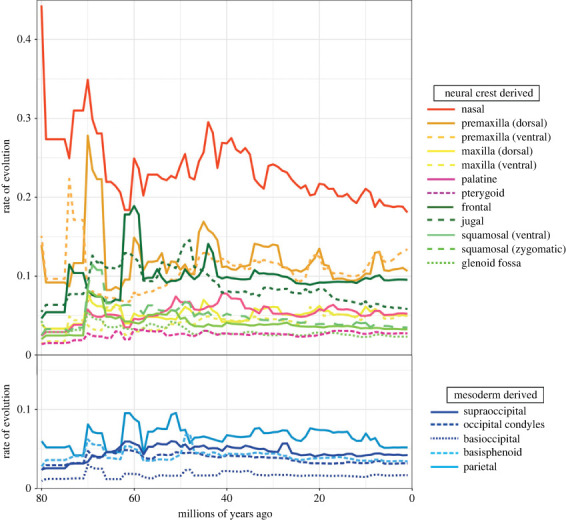
Rates of evolution for each cranial region plotted against time. Results from analyses with BayesTraits using phylogenetic PC scores representing 95% of the total variation, calculated separately for each cranial region. As a result, patterns through time, but not raw rate values, are directly comparable.

Plotting these rates of evolution from the BayesTraits analysis against time (figure 6) shows that some cranial elements, but not all, display the declining rates of evolution demonstrated by the entire skull (note that raw rate values are not directly comparable across regions as each was analysed separately) [2]. Under the assumption of a root age of mammals between 80–85 Ma (with the specific randomly selected tree displayed in figure 6 having a root age of 80.3 Ma), the nasals, premaxilla, maxilla, squamosal, basioccipital and glenoid fossa all show their highest rates of evolution during the initial diversification of placentals proximal to the end-Cretaceous mass extinction (figure 6). The nasals stand out as displaying particularly high early rates that fluctuate through time, with a peak just before the Cretaceous-Paleogene (K/Pg) boundary (66 Ma), followed by a steep decline. Rates increased steadily in the early Eocene, followed by general decline from the mid-Eocene through to the present day. The dorsal and ventral premaxilla show a similar pre-K/Pg peak and rapid decline, but then slowly rises in evolutionary rate through to the mid-Eocene and fluctuates around a stable and relatively high rate for the remainder of the Cenozoic. The dorsal and ventral maxilla also peak in rate proximal to the K/Pg boundary, but the ventral maxilla achieves similarly high rates again later in the Cenozoic and does not show as much variation in rates overall. By contrast, rates of evolution for the frontal, parietal and basisphenoid peak later in the Paleocene, while the pace of jugal evolution increases to a peak in the early to mid-Eocene, when the parietal also shows a second peak. The palatine similarly slowly increases in rate of evolution to the mid-Eocene, and then declines equally slowly. Finally, the pterygoid, occipital and basicranial elements generally show steady rates throughout the entirety of placental evolution (figure 6).

## 4. Discussion

What factors are most important for morphological diversification? Many deep-time studies focus on environment and ecology as the key forces governing selection [3,7,12,101–103], but developmental patterning and genetic interactions of traits generate the variation upon which natural selection can act and thus are also critical for shaping morphological evolution [14,17,45,57,59]. Discerning the relative importance of extrinsic and intrinsic factors is complicated, as hypotheses of developmental and functional patterning often overlap [25,66]. Indeed, the vertebrate skull is a developmental and functional composite that is formed from distinct cell populations (cranial neural crest and paraxial mesoderm) and serves multiple competing roles. In placental mammals, these roles include feeding and prey capture, housing and protecting neurosensory structures, locomotion such as head-driven burrowing, and supporting appendages for fighting and display [76,104–106]. These diverse functions are reflected in the wide variety of ecological niches that placentals have evolved into over the course of the Cenozoic era [1–3,94], but there is also substantial overlap in the form of the cranium across placental clades, either reflecting a high degree of conservation and/or convergence [2,36,73]. However, these areas of conservation or convergence are not evenly spread across the skull, nor are areas of divergence (figure 3; electronic supplementary material, figure S1), which may reflect different functions of skull regions or their divergent developmental origins, or both.

### (a) Macroevolutionary patterns of neural crest versus mesoderm-derived elements

The pluripotency of neural crest cells has often been suggested to drive morphological novelty, as in the formation of the vertebrate head and jaw [51,56], and more recently has been invoked as a driver of morphological variation in domesticated species [58–60] and sexually selected traits [57]. Recent studies of other vertebrate clades has also suggested that the greatest variation and fastest rates of evolution are observed in regions formed by cranial neural crest cells [14,19–21,35,61]. Here, our analyses of morphospace occupation for each of 17 cranial elements demonstrates that differentiation of placental mammal clades is most evident in the elements of anterior face and the cranial vault. The anterior face discriminates cetaceans, afrotherians and rodents from other placentals, while the cranial vault differentiates primates and whales from other clades (figure 3; electronic supplementary material, figure S1). Both of these regions are formed primarily, but in the case of the vault not entirely, by cranial neural crest cells. Specifically, while much of this differentiation is concentrated in the bones formed from CNC, the PM-derived elements of the posterior vault and occipital region, the parietal and supraoccipital bones in particular (electronic supplementary material, figures S1, S3 and S4), also show substantial change in some clades, such as rodents, whales and primates.

As hypothesized, the elements formed from CNC display the fastest and most volatile rates of evolution (figure 6). Indeed, the contrast in evolutionary tempo through time between elements originating from CNC cells versus those from PM is clear. With the single exception of the parietal, PM-derived elements show largely steady evolution through the Cenozoic (figure 6), without the rapid rises and falls observed for most CNC-derived regions. Of the CNC-derived regions, only the pterygoid shows similarly consistent rates of evolution through the Cenozoic, as is observed for most PM-derived basicranial and occipital elements.

The peaks in evolutionary rate observed in the CNC-derived elements, as well as the PM-derived parietal, likely reflect responses to environmental shifts driven by large-scale extrinsic events such as mass extinction and climate change. Linking these patterns to specific causes requires better resolution of the timing of placental evolution, but the attenuating evolution observed through the Cenozoic for the entire cranium [2] appears to be largely driven by the elements of the anterior face. The nasal, premaxilla and dorsal maxilla, as well as all regions of the squamosal, show the strongest pattern of highest rates in the Late Cretaceous to early Paleocene, which then decline or stabilize at lower levels through the Cenozoic, punctuated by smaller, later peaks. These elements, as well as the jugal, also show high rates in the middle Eocene, likely driven by major transitions in the early evolution of whales [8,11,107,108]. The ventral maxilla also achieves its highest rates early in placental evolution, but does not decline as steeply through the Cenozoic, with similarly high rates observed proximate to the Oligocene/Miocene boundary. By contrast, the elements of the vault, as well as the jugal, achieve their highest rates slightly later, with peaks in the mid to late Paleocene to early Eocene, rather than proximal to the K/Pg boundary. These shifts likely reflect the origin of Cetacea and Primates, both large-brained clades with distinctive vault morphology [11,107,109–111] (figure 3). While the frontal still displays an attenuating pattern after that peak, the parietal shows later peaks of equivalent magnitude, perhaps associated with the evolution and diversification of bats, which show rapid evolution of the parietal in their initial divergences (electronic supplementary material, figure S3) [9,10,12].

By contrast to the differences in evolutionary tempo between elements derived from CNC and those from PM, there is no significant difference in morphological disparity between these groups (table 1). While the most disparate elements are certainly derived from the CNC, several PM-derived elements, such as the parietal and supraoccipital, also display high disparity across placentals. Equally, CNC-derived regions such as the glenoid fossa, palatine and pterygoid display low disparity. Thus, our results suggest that, while elements derived from CNC cells show the greatest disparity and most responsive rates of evolution, those formed by PM are equally capable of achieving high disparity. The paraxial mesoderm may thus be favourably (from an evolutionary perspective) viewed as Aesop's mythical slow-and-steady tortoise, with neural crest cells taking the role of the erratic hare, both ultimately arriving at similar levels of variation.

### (b) Interrogating the usual suspects: allometric, phylogenetic and ecological effects on the evolution of cranial regions

Developmental origin is of course not the only factor impacting macroevolutionary trajectories of cranial regions. Size and phylogeny have long been identified as primary factors impacting skull evolution [23,112–118], and both are significantly associated with variation in shape for each cranial region studied here. Nonetheless, there is substantial range in their effects across the cranium, as may be expected given the potentially competing functions and pressures experienced by different cranial regions [4,73,105,112,113]. Phylogenetic signal is strongest in the anterior face and lowest in cranial base, contrary to long-standing assumptions of conservation of morphology in the basicranial region. The apparent conservativeness of this region has often been used to justify its heavy usage in phylogenetic analyses of mammals [119,120], although this has been contested in recent years [67,121,122]. Our results here confirm that the basicranium does not appear to be phylogenetically conservative across placentals as a whole, although it also is not a particularly disparate or fast evolving region across placentals, contrary to previous analyses showing faster rates of basicranial evolution in Carnivora [66,67].

By contrast to the regional distribution of phylogenetic signal, size had the strongest effect in the vault, followed by the face. This is perhaps expected, as the allometry of the brain and face in mammals and other big-brained vertebrates has long been a topic of debate [23,112,114,123], driven in large part by a clear negative allometry of brain size and body size that is likely driven by energetic and/or developmental costs of large brains [111,124–127]. A long-standing hypothesis posits that the face and braincase should show opposing patterns of allometry, with the brain's negative allometry with size buffered by the face's positive allometry, which together result in a near-geometric scaling of the skull overall [112–114,123]. The results here support the hypothesis that cranial allometry is dominated by these two regions, and it is noteworthy that the strongest effect is observed in the braincase, though establishing which region drives this pattern of cranial allometry requires explicit analysis [113].

The two ecological factors considered here, diet and locomotion, have previously been shown as significantly associated with cranial variation for the whole skull [2,73,102,128–138]. Here, we show that these associations are supported in roughly half of the cranial elements, with diet influencing slightly more elements, and with stronger effect, than does locomotion [73,137]. The strongest dietary signal was observed in the midface, palate, zygomatic and occipital regions, but perhaps surprisingly not the anterior face. Locomotory signal was similarly diffuse, in anterior and midface, zygomatic and occipital region, but consistently with a lower effect size than diet or size. These associations of locomotion with specific regions likely relates to feeding, prey capture, head orientation, and jaw and neck muscle insertions, and thus there may be an interaction between diet and locomotion [73,139,140]. Although we were not able to assess this here as many intersecting bins have no representatives (e.g. there are no arboreal bulk invertivores), the interaction of diet and locomotion in placental skull evolution warrants further investigation.

It may be considered unsurprising that diet has a strong effect on many cranial regions, equal in some cases to that of size, given the key role that the skull plays in food acquisition and processing in most mammals. Nonetheless, this result contrasts with similar-scale studies of birds [103,141], where diet is consistently only weakly associated with shape of the skull and that of most skull regions. Squamates, however, similarly to placentals, show significant and stronger associations of diet and locomotion (as well as habitat) with the shape of cranial regions [19]. However, the associations of diet are limited to the anterior face for lizards and the suspensorium of snakes, while locomotion is significantly associated with all cranial regions. Thus, these results suggest that diet may be a more significant driver of cranial variation in mammals than in other amniote clades, while locomotion is possibly less important as a driver than seen in other amniotes, particularly clades with a higher proportion of burrowing forms. We hypothesize that the stronger impact of diet on the shape of many cranial regions, relative to that observed in other amniotes, likely reflects the increased complexity of food processing in mammals. Many of the key innovations in early mammal evolution, including tribosphenic teeth, heterodonty, diphyodonty and the single jaw bone, relate to the evolution of a more efficient masticatory system to support a higher metabolism [142–145], with a resulting complexity in both tooth structure and masticatory apparatus [143,145–147] that sets mammals apart from other terrestrial vertebrate clades. Focusing in on the specific regions that show the strongest associations with diet (midface, palate, zygomatic and occipital regions), it is evident that these associations are not dominated by any one dietary niche or clade (electronic supplementary material, figures S3 and S4). Rather, as detailed in the Results, numerous clades and dietary niches show accelerated evolution in each of these regions, suggesting that the pattern observed reflects a more general association of diet and cranial region shape across placentals and not a strong effect of any one unusual group.

Rates of cranial evolution may also be influenced by ecology even where cranial morphological variation does not show a clear association [20,21,103]. Here, we found that bulk invertivores and especially piscivores show the fastest rates of evolution across the palate, anterior face, parietal and occipital condyles (figure 4). Whales dominate these two dietary categories (although not exclusively for piscivores), and shifts in these regions likely relate to changes involved in suction feeding and cranial telescoping [11,109,148]. However, non-whale aquatic mammals, including piscivorous pinnipeds also show shifts in their nares, rostrums and palates, associated with respiration, sexual dimorphism and feeding behaviour [72]. Herbivores show the fastest rates of evolution in anterior face and zygomatic region, reflecting elongation of the face and modification of the premaxilla for either ever-growing incisors or entire loss of these teeth [146,149–152], as well as presence of a complete postorbital bar in many herbivores, such as equids, some artiodactyls, and primates [116,135,149]. By contrast, carnivores show the fastest evolution for the midface and vault, with the latter potentially reflecting increased attachment area for the temporalis muscle [73,102,137,138,153–155]. Insectivores show high rates in the posterior skull, likely relating to adaptations for fossoriality in many taxa [80,106,146,156].

With regards to locomotion, aquatic taxa, as expected, show the fastest rates in most cranial modules, but semi-aquatic taxa also show high rates in anterior face, perhaps reflecting modification of the nares for thermoregulation and respiration during prolonged periods of swimming in these taxa [157]. Fossorial and volant taxa both show fast rates in the posterior skull, likely for different reasons. Fossorial taxa often have modified vault shapes related to burrowing behaviour [80,106,156], while volant taxa have unusual modifications of the vault and occipital region likely relating to neurosensory demands of echolocation and aerial manoeuvrability [12,158]. Finally, arboreal taxa show rapid evolution of the zygomatic region, likely reflecting modifications of the jugal, exemplified in the postorbital bar and plate of Primates, and related to stereoscopy and emphasis on vision to navigate the complex three-dimensional arboreal environment [129–132,159].

These patterns of evolutionary rates for specific ecological groups are supported by comparisons across clades, where rates of evolution for cranial regions reflect the established morphological adaptations associated with placental orders. From the rapid palatal and vault evolution observed in Chiroptera [12,98–100] to the rapid evolution of the frontal in the highly ornamented brontotheres [160] and in litopterns, which, similarly to whales, have posteriorly shifted nares and deeply grooved frontal bones [96,161], these results demonstrate that cranial regions show distinct macroevolutionary patterns that reflect the diverse ecologies and adaptations of placentals. Combined, these results further demonstrate that there is no clear differentiation in association with ecology, irrespective of phylogenetic relationships, segregating elements derived from CNC or PM. Elements derived from CNC cells show the fastest rates of evolution, but ecological signal is equally pronounced in bones derived from either cell population. Moreover, disparity, while highest in the CNC-derived elements, does not differ significantly between the two groups. Indeed, the posterior vault and basicranium clearly evolve specializations for volant and fossorial lifestyles, as readily as the nasal does for an aquatic one. Thus, while elements originating from cranial neural crest cells may be more responsive to selective pressure and evolve more quickly, this does not necessarily translate to greater disparity or ecological signal, or to less association with phylogenetic relatedness, than is observed in elements derived from paraxial mesoderm.

Just as taxonomic diversity and morphological disparity do not necessarily correspond on macroevolutionary timescales [162], patterns for evolutionary rates and morphological disparity may differ for numerous reasons, including selective extinction, ecological convergence, competition or developmental constraints. Integration among traits can represent a constraint on variation of individual traits, and recent theoretic and empirical work on phenotypic integration has described the ‘fly in the tube’ model [68], in which strong covariation of traits constrains variation, and therefore disparity, but not necessarily rate of evolution. Our results here suggest a similarly divergent, albeit reversed, impact of mesenchymal cell populations on evolutionary rates and morphological disparity in the placental skull. Specifically, developmental origin may influence the evolutionary tempo of cranial elements, but it does not constrain their capacity for functional and ecological specialization.

## Data Availability

Three-dimensional meshes for all specimens are available for free download on Phenome10k.org and/or Morphosource.org, unless specifically restricted by specimen repositories. All data and novel code, as well as interactive PCAs, are available at https://github.com/anjgoswami/Goswami_et_al_Placental_evolution_2022. All specimen and species details, including repository information and trait data, are provided in electronic supplementary material, table S1. The data are provided in electronic supplementary material [163].
